# A highly sensitive printed humidity sensor based on a functionalized MWCNT/HEC composite for flexible electronics application†

**DOI:** 10.1039/c9na00179d

**Published:** 2019-04-15

**Authors:** Vikram S. Turkani, Dinesh Maddipatla, Binu B. Narakathu, Tahseen S. Saeed, Sherine O. Obare, Bradley J. Bazuin, Massood Z. Atashbar

**Affiliations:** Department of Electrical and Computer Engineering, Western Michigan University 4601 Campus Drive Kalamazoo Michigan-49008 USA vikramshreeshail.turkani@wmich.edu +1 269 276 3148; Department of Chemistry, Western Michigan University 1903 Western Michigan Avenue Kalamazoo Michigan-49008 USA

## Abstract

A novel functionalized multi-walled carbon nanotube (FMWCNT)/hydroxyethyl cellulose (HEC) composite-based humidity sensor was successfully developed for humidity monitoring applications. FMWCNTs were synthesized by covalently functionalizing multi-walled carbon nanotubes (MWCNTs) in a mixture of sulfuric and nitric acid to enhance their hydrophilicity. The FMWCNTs were characterized using transmission electron microscopy, Raman spectroscopy, Fourier transform infrared spectroscopy and dispersion analysis to verify the presence of functional hydroxyl and carboxyl groups. A FMWCNT/HEC (1 : 6 w/w) composite ink was formulated using the solution blending technique with 2.5 wt% FMWCNTs. A multi-layered humidity sensor was fabricated using additive print manufacturing processes on a flexible polyethylene terephthalate (PET) substrate. Screen printing and gravure printing processes were used to deposit the bottom silver (Ag) electrode and FWMCNT/HEC sensing layers, respectively. The capability of the fabricated humidity sensor was investigated by measuring its resistive response towards relative humidity (RH) varying from 20% RH to 80% RH. As the RH was increased from 20% RH to 80% RH in steps of 10% RH at 25 °C, it was observed that the resistance of the printed sensor increased linearly. The printed sensor demonstrated resistance changes as high as ≈290% at 80% RH, when compared to its base resistance at 20% RH. A sensitivity and a response time of 0.048/%RH and ≈20 s were obtained for the printed sensor, respectively. The results thus demonstrated the feasibility of employing additive print manufacturing processes to develop a highly sensitive sensor for humidity monitoring applications.

## Abstract 2

Fully printed, functionalized multi-walled carbon nanotube (FMWCNT)/hydroxyethyl cellulose (HEC) composite-based humidity sensor.

## Graphical abstract

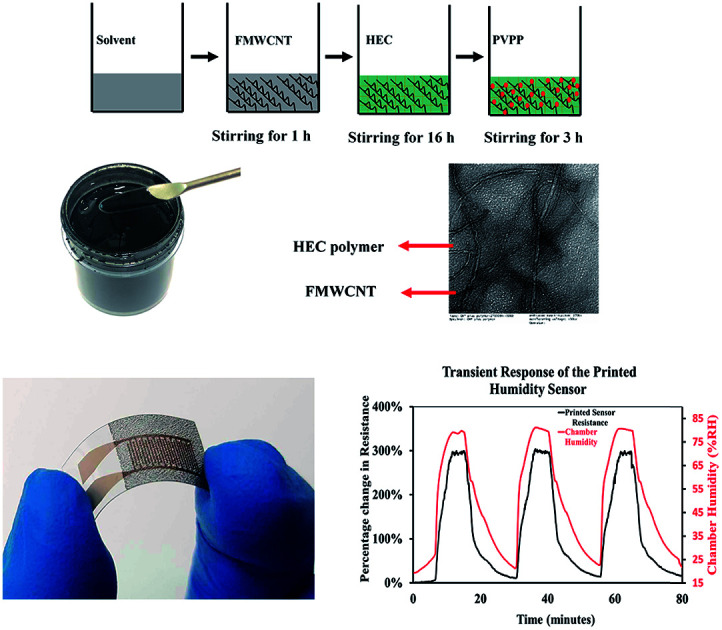

## 1. Introduction

Over the years, the need for monitoring humidity in the automobile, medical and food industries has become a growing requirement.^1–4^ Further, the maintenance of ambient conditions within human habitats for optimum comfort has often been influenced by humidity, thus making it one of the vital parameters that needs to be monitored.^5^ Owing to this importance, humidity measurement has been extensively studied and, accordingly many researchers have developed various types of humidity sensors based on different sensing principles and diverse hygroscopic materials.^6,7^ Humidity is measured as absolute humidity (AH) and relative humidity (RH) which provide the true value and relative value (in terms of pressure) of water vapor content in a given volume of air, respectively.^6^ RH based sensors are more commonly used for monitoring humidity due to their ease-of-use and cost-efficiency.^6^ They have been extensively used in applications involving indoor air quality, human comfort issues and research laboratories.^6^ Therefore, the development of humidity sensing systems that employ novel RH sensors is of utmost importance.

Resistive type RH humidity sensors are the most commonly implemented type in the industry, as they are much easier to integrate and use relatively simpler electronics to monitor and control ambient humidity.^8^ Most of the resistive type humidity sensors are fabricated by coating hygroscopic polymers including polyimide,^8–10^ co-polymerized PMMA/PMAPTAC^12^ and polyelectrolyte^13^ as the humidity sensitive films.^12^ However, these devices are typically slow and suffer from a relatively longer response time (105 s).^14^ Moreover, these sensors have lower humidity detection limits (30–42% RH), making them impractical for use in many industrial and domestic applications.^8,15^ Therefore, research has been focused on the development of novel materials for resistive type humidity sensors to overcome the drawbacks associated with polymeric based resistive type humidity sensors. Owing to their large surface area to volume ratio and hollow cylindrical nanostructure,^16^ multiwalled carbon nanotubes (MWCNTs) have been extensively employed for sensing various molecules adsorbed on their surface.^17,18^ Research has been reported on MWCNT based resistive type humidity sensors. Liu *et al.* reported a resistive type humidity sensor based on pristine MWCNT networks and demonstrated its capability to sense a broad range of relative humidities (25% RH to 85% RH) with good linearity and an excellent response time of 3 s.^19^ However, this device suffered from a low sensitivity of 0.5%/%RH towards humidity sensing.^19^ To improve the sensitivity of the MWCNT based humidity sensor, Cao *et al.*^20^ chemically treated the surface of MWCNTs and compared the humidity sensing characteristics of both chemically treated and untreated MWCNTs. It was observed that the former had greater sensitivity than the latter due to the presence of hydrophilic functional groups on the surface of the chemically treated MWCNTs.^20^ Thus, MWCNTs are promising as humidity sensitive materials. However, more efforts are needed towards the development of novel MWCNT based, highly sensitive, resistive type humidity sensors.

Conventionally, humidity sensors have been manufactured on rigid structures like glass or ceramics using techniques such as photolithography and sputtering.^21,22^ These techniques are relatively expensive and time consuming as they require high vacuum, large power density, and a high temperature environment. Moreover, the rigid nature of the substrates prevents their use in applications which require mechanical flexibility and conformal form factors. To overcome these limitations, additive print manufacturing processes such as screen, inkjet, gravure and flexography can be used for the development of cost efficient, flexible and conformal humidity sensors. These additive print manufacturing processes have already enabled an emerging field called flexible hybrid electronics to develop novel, cost-efficient electronic devices for applications that demand flexibility and conformability. Accordingly, many researchers have actively employed these processes for the development of RFID tags,^23,24^ sensors,^25–34^ solar cells,^35,36^ antennas^37^ and circuits.^38,39^ In a recent study, a CNT based humidity sensor fabricated using two printing processes, screen and gravure printing, was reported.^40^ In addition, Xie *et al.* also reported an inkjet printed MWCNT based humidity sensor.^41^ Although the printed sensors in both the cases responded towards ambient humidity, there is still a need to improve their sensitivity. Therefore, the use of additive print manufacturing processes to develop highly sensitive humidity sensors employing MWCNTs and/or MWCNTs/polymer composites is envisioned to advance the field of humidity sensing.

In this work, a multi-layered functionalized multi-walled carbon nanotube (FMWCNT)/hydroxyethyl cellulose (HEC) composite-based humidity sensor was developed using additive print manufacturing processes on a flexible polyethylene terephthalate (PET) substrate. MWCNTs were subjected to acid functionalization in a mixture of sulfuric and nitric acid. Transmission electron microscopy, Raman spectroscopy and Fourier transform infrared spectroscopy were performed to verify the functionalization of MWCNTs. Screen printing, a push through process was employed to deposit the electrodes in an interdigitated structure. The FMWCNT/HEC composite ink was formulated and deposited on the electrodes as the humidity sensing layer using gravure printing. The performance of the printed humidity sensor was investigated by measuring its resistive response towards relative humidity (RH) varying from 20% RH to 80% RH at a constant temperature of 25 °C.

## 2. Experimental

### 2.1 Chemicals and materials

A flexible PET film (MELINEX® ST730) from DuPont Teijin Films was used as the substrate. Conductive Ag ink (AG-800) from Applied Ink Solutions was used for the electrodes. The conductive filler for the sensing layer was MWCNTs, with diameters and lengths of 20–30 nm and 10–30 nm, respectively (US Research Nanomaterials, Inc., US4039, purity > 95 wt%). Analytical grade 95% sulfuric acid (H_2_SO_4_) and 70% nitric acid (HNO_3_) from Sigma-Aldrich Chemical Company were used for the functionalization of the MWCNTs *via* acid treatment. Hygroscopic HEC (Cellosize™ Hydroxyethyl Cellulose EP-09, DOW chemical company) and polyvinylpolypyrrolidone (PVPP) polymer (ViViPrint™ 540, Ashland Performance Materials) were used as the polymer matrix and binder in the FMWCNT/HEC ink, respectively. Acetone, from Sigma-Aldrich Chemical Company, and de-ionized (DI) water were used as the cleaning solvents for the Ag and FMWCNT/HEC inks, respectively. The electrical connections for the printed sensor were made using contact flat flex interconnects (Model no. 1-88997-2) from TE Connectivity AMP Connectors. The headers of these interconnects were connected using male to female jumper wires (Model no. 1568-1511-ND) from SparkFun Electronics.

### 2.2 Functionalization of MWCNTs

The MWCNTs were subjected to a covalent functionalization process which involves surface modification of the MWCNTs by incorporating hydrophilic substituents onto the exterior MWCNT sidewalls^42^ (Fig. S1†). The aim of the functionalization process was to improve the hydrophilicity of MWCNTs besides aiding their dispersion in aqueous medium.^43,44^ 2 g of MWCNTs were added to a 3 : 1 (v/v) mixture of H_2_SO_4_ and HNO_3_, respectively and the mixture was refluxed at 140 °C using continuous magnetic stirring for 1 hour.^11^ The mixture was allowed to cool down to room temperature after the reflux. Then, the obtained mixture was titrated against NH_3_OH until a pH of 5.5 was attained.^44^ The neutralized solution was then vacuum filtered to separate the FMWCNTs using 0.2 μm pore size polytetrafluoroethylene membrane filters. Finally, the FMWCNTs were washed with DI water several times and dried for 12 hours at 140 °C.

### 2.3 Characterization of the FMWCNTs

Transmission electron microscopy (TEM) and Raman spectroscopy were used to obtain morphological information and to determine the degree of functionalization, respectively, of the MWCNTs before and after acid treatment. A JEOL 100 CXII TEM with an accelerated voltage of 200 kV was used to record the TEM micrographs and analyze the morphology of MWCNTs. Two samples were prepared by dispersing the MWCNTs in ethanol and the FMWCNTs in DI water. The prepared samples were dropped on a carbon coated 200-mesh copper grid for imaging.

The experimental setup for Raman spectroscopy is shown in Fig. S2.† The MWCNT and FMWCNT samples were placed in a sample holder (PA-SH02, Inphotonics Inc). A laser source in the near infrared region with a wavelength of 785 nm was used to excite the samples using a Raman probe from Inphotonics Inc. with an integration time of 3 seconds at 300 mW. Raman analysis was performed for both the MWCNTs and the FMWCNTs, and a comparison was made to obtain the degree of functionalization of the MWCNTs after acid treatment.^44^ A spectrometer (QE 6500) from Ocean Optics Inc. was used to obtain the Raman spectra. The Raman spectra were then analyzed using Spectra Suite Software from Ocean Optics Inc.

Fourier transform infrared spectroscopy (FT-IR) was performed to verify the presence of hydrophilic constituents on the surface of the FMWCNTs. Two samples, one with MWCNTs and another with the FMWCNTs, were prepared by mechanically mixing them with potassium bromide powder. The mixtures were pressed into discs and were placed in a ThermoFisher Nicolet iS5 spectrometer. The FT-IR spectra were analyzed using OriginPro software.

The dispersion analysis was performed on both MWCNTs and FMWCNTs to compare their suspension stability in DI water, thereby demonstrating the efficiency of functionalization.^44^ 0.125 g (0.5 wt%) of MWCNTs and FMWCNTs were dispersed in 25 mL of DI water. The solutions were stirred vigorously for 60 minutes and held for 5 days, which is a sufficient duration required to formulate the ink and fabricate the sensor.

### 2.4 FMWCNT/HEC ink formulation

**Fig. 1:**
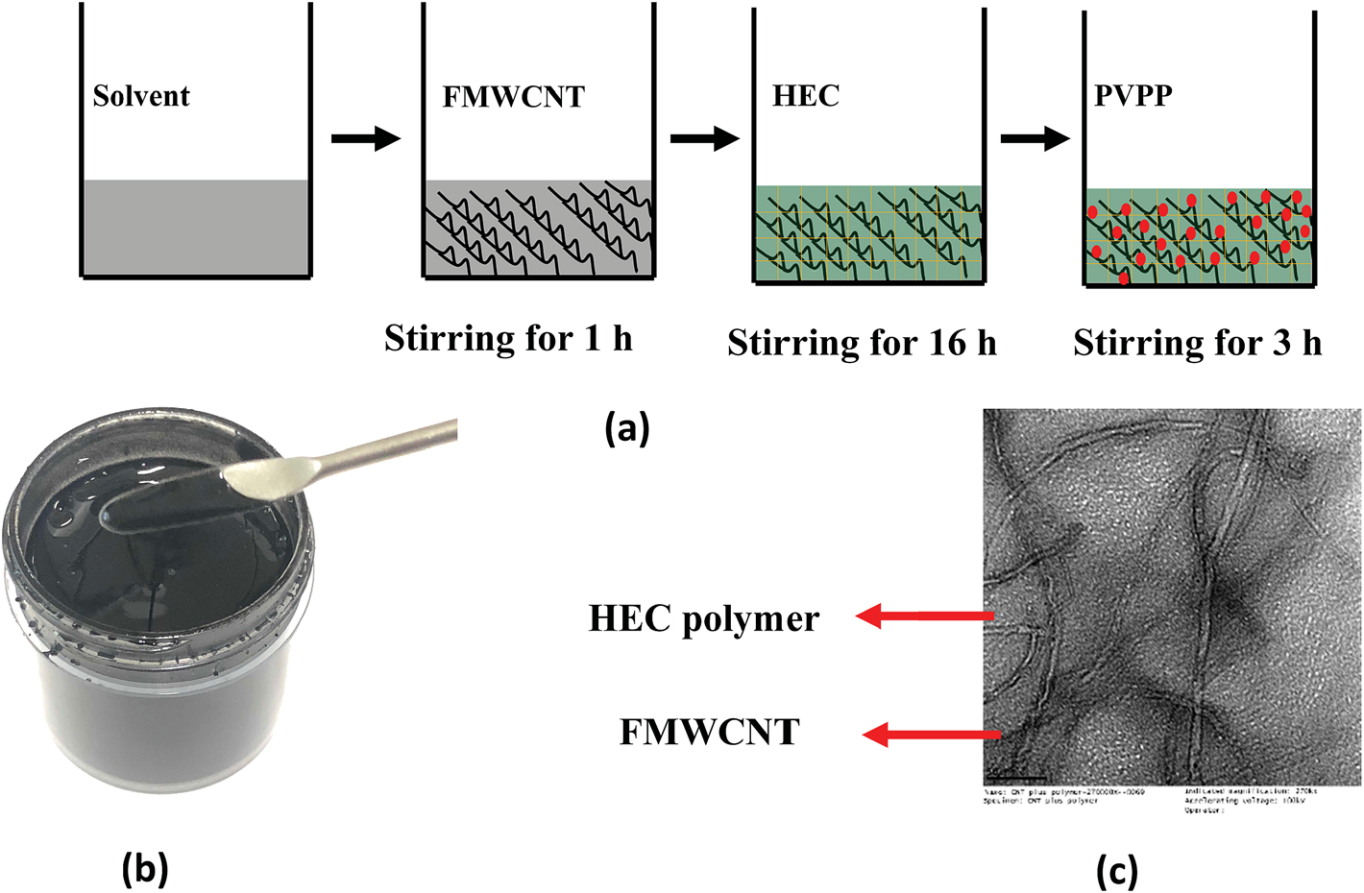
FMWCNT/HEC ink formulation: (a) steps involved in ink formulation, (b) photograph of the FMWCNT/HEC ink and (c) the TEM image of the FMWCNT/HEC ink.

The FMWCNT/HEC ink was formulated using DI water as the solvent. Fig. 1(a) shows the steps involved in the ink formulation. Initially, hydrophilic FMWCNTs (1.25 wt% and 2.5 wt%) were added into the solvent, followed by magnetic stirring for 1 hour to ensure proper dispersion. General dispersion techniques such as bath and probe sonication were not considered as these techniques would result in excessive evaporation of the solvent.^45^ The FMWCNT/HEC composite for the ink was prepared using the solution blending technique.^11^ FMWCNT/HEC composites with varying ratios (1 : 4 w/w and 1 : 6 w/w) were prepared for studying the humidity response. In addition to being a hygroscopic polymer, HEC is also an efficient dispersant in the ink system as it is completely water soluble and swells in the presence of water, thereby preventing agglomeration between the FMWCNTs.^11,46^ The mixture was stirred for 16 hours to obtain homogeneity in the composite. Finally, PVPP was added and magnetically stirred for 3 hours to form the FMWCNT/HEC ink (Fig. 1(b). PVPP was chosen as a suitable binder due to its swelling behavior in water and its loading was restricted to 50 wt% FMWCNTs, maintaining a binder to filler ratio of 1 : 2. The TEM image of the FMWCNT/HEC ink (Fig. 1(c)) shows the FMWCNTs embedded in the HEC polymer matrix.

### 2.5 Assessment of the print compatibility

**Fig. 2:**
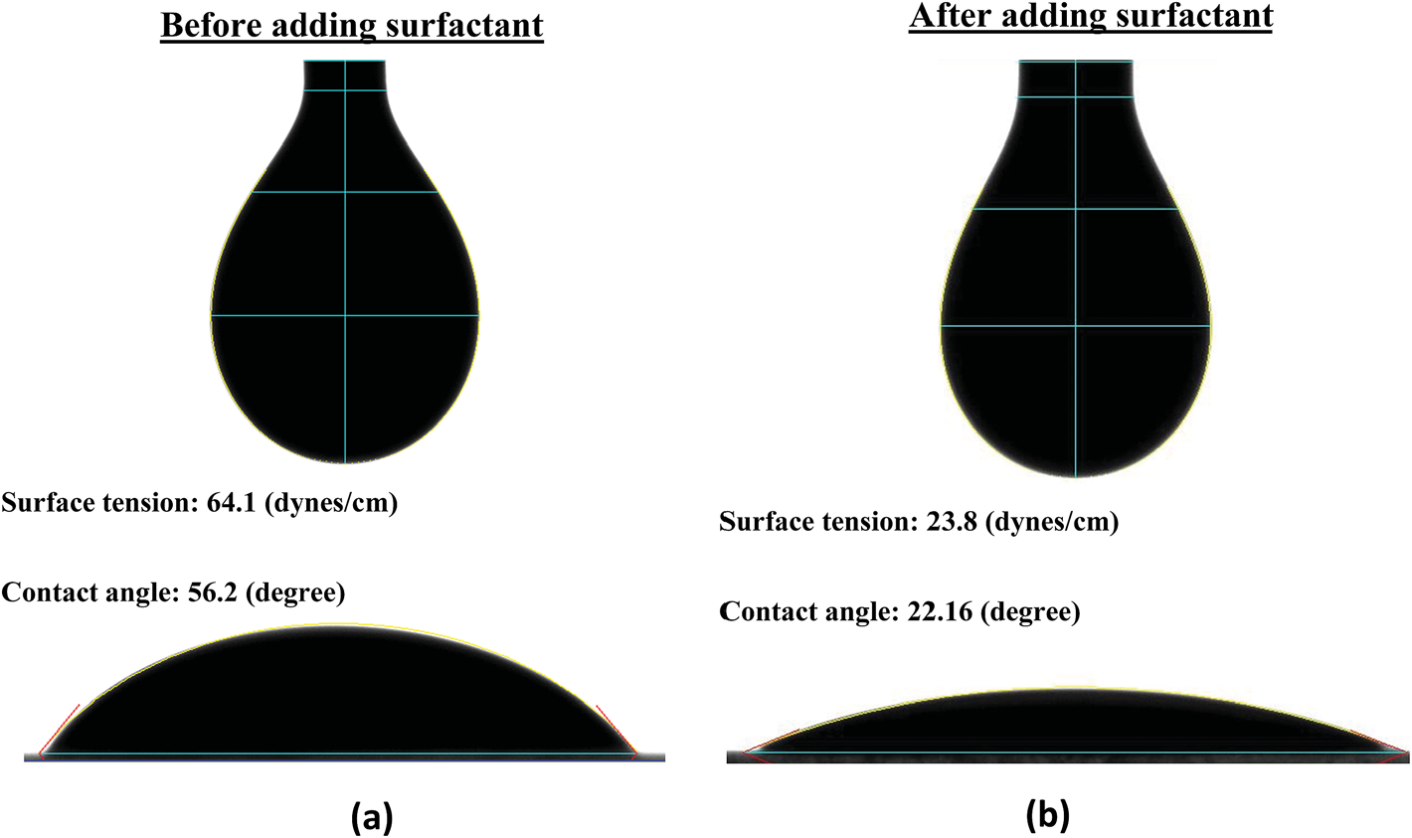
Surface tension of the FMWCNT/HEC ink (a) before and (b) after adding the surfactant.

The surface tension (ST) and contact angle (CA) of the formulated FMWCNT/HEC ink were measured to be 64.1 (dynes cm^−1^) and 56.2 (degrees) with a goniometer (First Ten Angstroms FTA-200) using the pendant drop method^49^ and sessile drop method,^50^ respectively (Fig. 2(a)). The surface energy (SE) of the PET was also measured to be 45.4 (dynes cm^−1^) with the goniometer using the Owens Wendt method.^48^ It was observed that the ST of the ink was 18.7 units greater than the SE of the substrate. However, a good print compatibility between the printed layer and the substrate is obtained when the SE of the substrate is greater than the ST of the ink.^47^ This is because the SE of the substrate, which is the energy due to the intermolecular forces, should be able to break the resistance of the ink and to deform it into a new surface.^47^ The resistance offered by the ink, due to attractive intermolecular forces formed on its interfacial surface, is the ST of the ink.^47^ Therefore, 1 wt% Ecosurf™ (Dow Chemical Company) surfactant was added to the prepared FMWCNT/HEC ink and was magnetically stirred for 3 hours for reducing its ST. It was observed that the ST decreased to 23.8 (dynes cm^−1^), which is 21.6 units less than the SE of the substrate (Fig. 2(b)). Thus, it was concluded that the FMWCNT/HEC ink was print compatible with the PET substrate.

### 2.6 Humidity sensor fabrication

**Fig. 3:**
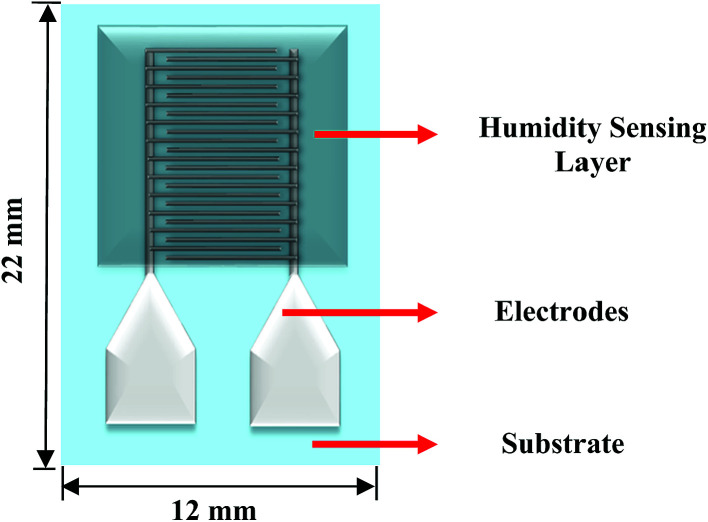
Schematic of the humidity sensor (not to scale).

Fig. 3 shows the schematic of the humidity sensor. It consists of three layers: substrate, electrodes and a sensing layer. The sensor was designed with an overall dimension of 22 mm × 12 mm in Adobe Illustrator® design software. The sensor consists of a pair of electrodes, with 24 interdigitated (IDT) fingers that are 5200 μm long and 200 μm wide, with a pitch of 400 μm. The sensing layer was designed to be 14 mm × 12 mm.

**Fig. 4:**
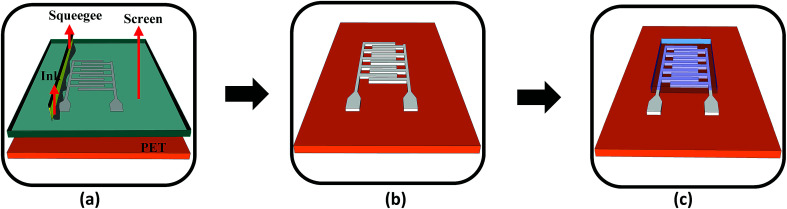
(a) Screen printing of Ag ink on the PET substrate, (b) screen printed IDTs and (c) gravure printing of the MWCNT/HEC ink on the electrodes (not to scale).

**Fig. 5:**
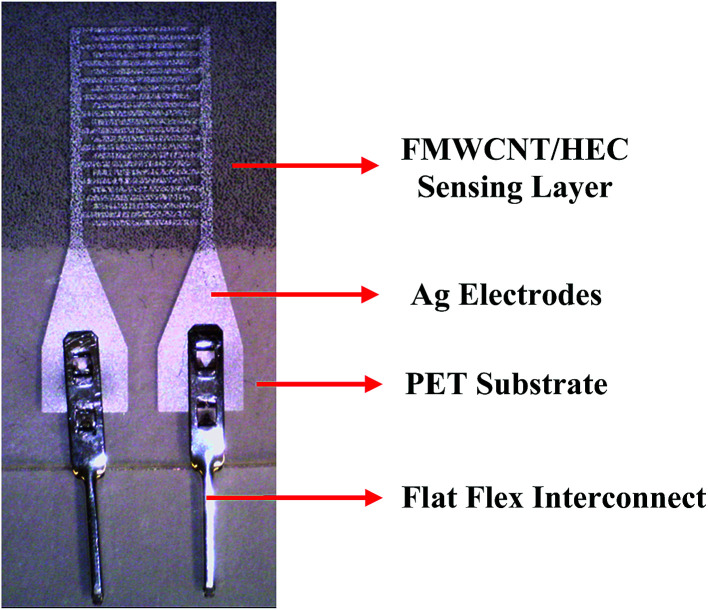
Photograph of the printed sensor.

The fabrication of the MWCNT/HEC based humidity sensor was performed on the flexible PET substrate using screen and gravure printing processes. Fig. 4 shows the fabrication steps of the humidity sensor. Initially, the surface of the PET substrate was cleaned with isopropyl alcohol (IPA) and was heated at a temperature of 80 °C for 2 hours on a VWR® Signature 810 hot plate to remove any organic impurities present on the surface of the substrate. Then, a semi-automatic screen printer (AMI MSP 485) was used to deposit Ag ink on the PET. A stainless steel screen, fabricated at Microscreen®, with 325 mesh count, wire diameter 28 μm, mesh angle 22.5° and 12.7 μm thick MS-22 emulsion was used for screen printing. The printed Ag ink was thermally cured in a VWR 1320 temperature controlled oven at 135 °C for 5 minutes to form the Ag based IDTs. Finally, a laboratory scale gravure press (K-Printing Proofer) was used to deposit the FMWCNT/HEC ink on the IDTs as a humidity sensitive layer. An electromechanically engraved gravure plate, from IR Engraving LLC, with a 200-line screen (LS) and 45° cell angle was used for gravure printing. The printed FMWCNT/HEC ink was thermally cured at 110 °C for 3 minutes. A photograph of the fabricated humidity sensor is shown in Fig. 5.

### 2.7 Surface characterization of the printed layers

**Fig. 6:**
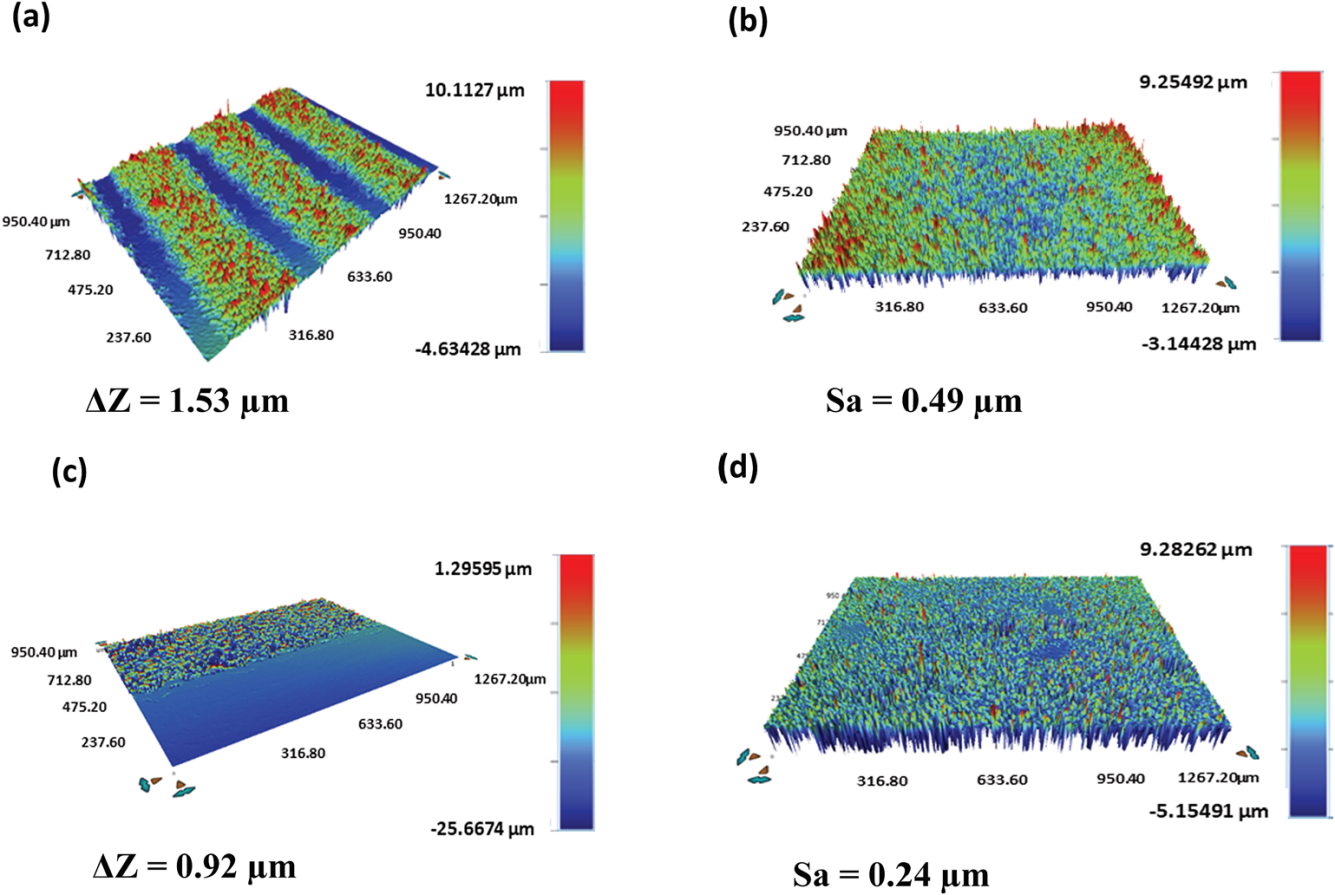
3D output of the vertical scanning interferometry showing the (a) thickness and (b) roughness of silver electrodes and the (c) thickness and (d) roughness of the FMWCNT/HEC sensing layer.

A Bruker Contour GT-K vertical scanning interferometer was used to characterize the thickness and roughness of the printed layers (Fig. 6). An average thickness (Δ*Z*) of 1.49 ± 0.05 μm and 0.94 ± 0.03 μm and an average surface roughness (Sa) of 0.52 ± 0.04 μm and 0.28 ± 0.05 μm were obtained for the Ag electrodes and FMWCNT/HEC sensing layer, respectively.

### 2.8 Experimental setup

**Fig. 7:**
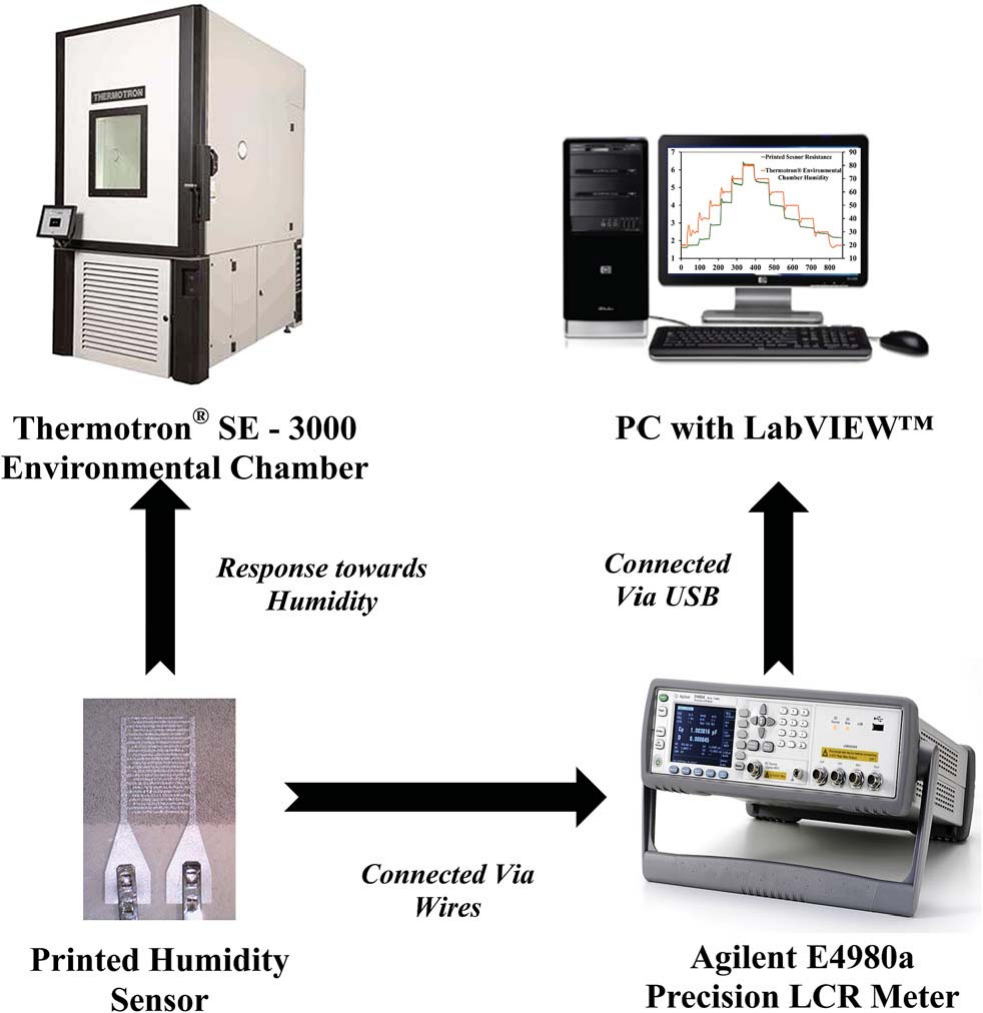
Experimental setup.

The experimental setup for investigating the response of the printed humidity sensor towards varying relative humidity (%RH) is shown in Fig. 7. Electrical connections to the printed sensor were made using the FFCs. The printed sensor was subjected to relative humidity varying from 20% RH to 80% RH, in steps of 10% RH, at a constant temperature of 25 °C in a Thermotron® SE 3000 environmental chamber. The chamber was equipped with a Thermotron® 8800 data acquisition (DAQ) system for controlling, monitoring, graphing and reporting environmental chamber data. The relative humidity and temperature of the chamber were recorded using an integrated humidity sensor HUMICAP 180 from Vaisala and a T-type (Copper/Constantan) thermocouple (T-20 B/W), respectively. During the experiment, an Agilent E4980A precision LCR meter, controlled by a custom-built LabVIEW™ program on a PC, was used to record the resistance of the printed humidity sensor at an operating frequency of 1 kHz and an applied voltage of 1 V.

## 3. Results and discussion

### 3.1 Transmission electron microscopy (TEM)

**Fig. 8:**
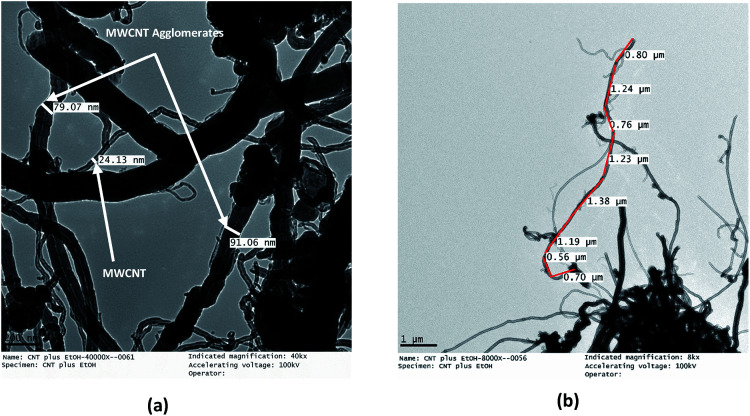
TEM micrographs of MWCNTs showing (a) a diameter of ≈20–30 nm for the MWCNTs and a diameter of ≈80–90 nm for the MWCNT agglomerates, and (b) a length of ≈8–17 μm for the MWCNTs.

The TEM micrographs of the MWCNTs and FMWCNTs, which indicate a tube-like morphology, are shown in Fig. 7 and 8, respectively. It was observed that the ends of the nanotubes were closed due to the presence of amorphous carbonaceous impurities around the MWCNTs (Fig. 7). Large agglomerates (≈80–90 nm) of MWCNTs were present due to van der Waal's forces of attraction.^20,42^ The diameter and length of the MWCNTs were measured to be in the range of ≈20 nm to 30 nm (Fig. 8(a)) and ≈8 μm to 17 μm (Fig. 8(b)), respectively.

**Fig. 9:**
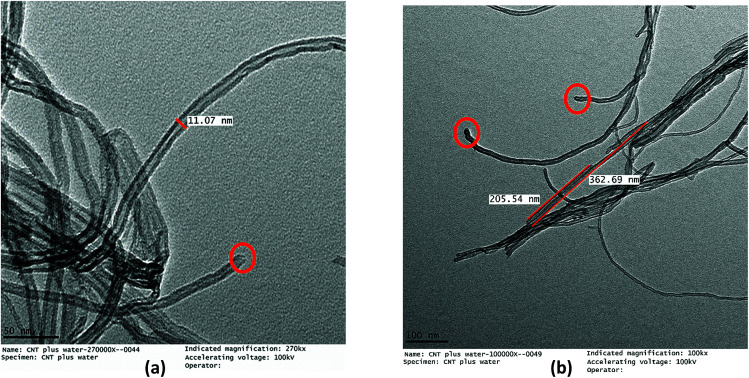
TEM micrographs of FMWCNTs showing (a) a diameter of 10–20 nm (marked in red circles) and (b) a length of 0.1–1 μm.

The acid treatment of the MWCNTs enhanced the arrangement of tubes resulting in less agglomeration of the MWCNTs and removal of the carbonaceous impurities due to which the ends of the MWCNTs opened (marked with a red circle) (Fig. 9). This phenomenon is consistent with results obtained by Cao *et al.* and Rahmam *et al.*^20,43^ It was observed that, after the acid treatment, the diameter and length of the FMWCNTs were in the range of 10 nm to 20 μm and 0.1 μm to 1 μm, respectively. The reduction in the length of the FMWCNTs can be attributed to the oxidative etching during the acid treatment. In addition, the acid treatment also introduced “defects” on the outermost walls of the MWCNTs, which indicates the presence of functional groups that make the FMWCNTs more hydrophilic^51^ (Fig. S3†).

### 3.2 Raman spectroscopy

**Fig. 10:**
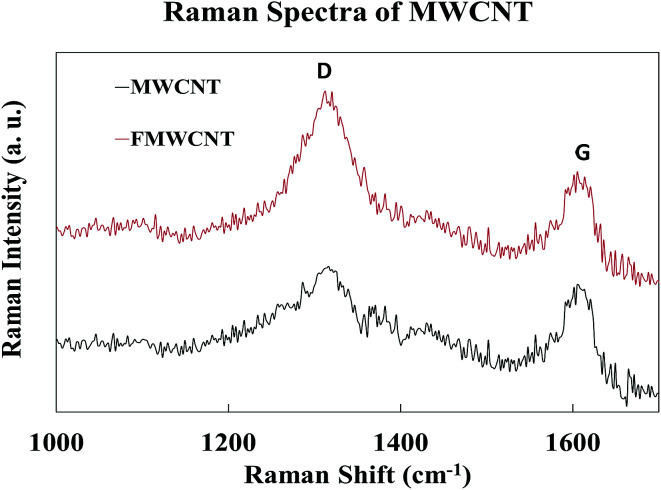
Raman spectra of MWCNTs and FMWCNTs.

Fig. 10 shows the Raman spectra of MWCNTs and FMWCNTs. Two characteristic peaks of MWCNTs corresponding to the graphite band (G-band) and the disorder/defect band (D-band) were observed in the high-frequency region of the spectra for both MWCNTs and FMWCNTs; at ≈1320 cm^−1^ and ≈1613 cm^−1^, respectively.^44,52^ The relative intensity (*I*_(D)/(G)_), which is the ratio of the D-band intensity (*I*_(D)_) to the G-band intensity (*I*_(D)_), provides information on the degree of functionalization. *I*_(D)/(G)_ values of 1.05 and 1.33 were calculated for the MWCNTs and FMWCNTs, respectively, indicating that there was a 27% increase in the number of defective sites on the surface of the FMWCNTs. The increase in the number of defects can be attributed to the acid functionalization process that breaks the bond between the C=C atoms and inserts functional groups.^44,53^

### 3.3 Fourier transform infrared spectroscopy

**Fig. 11:**
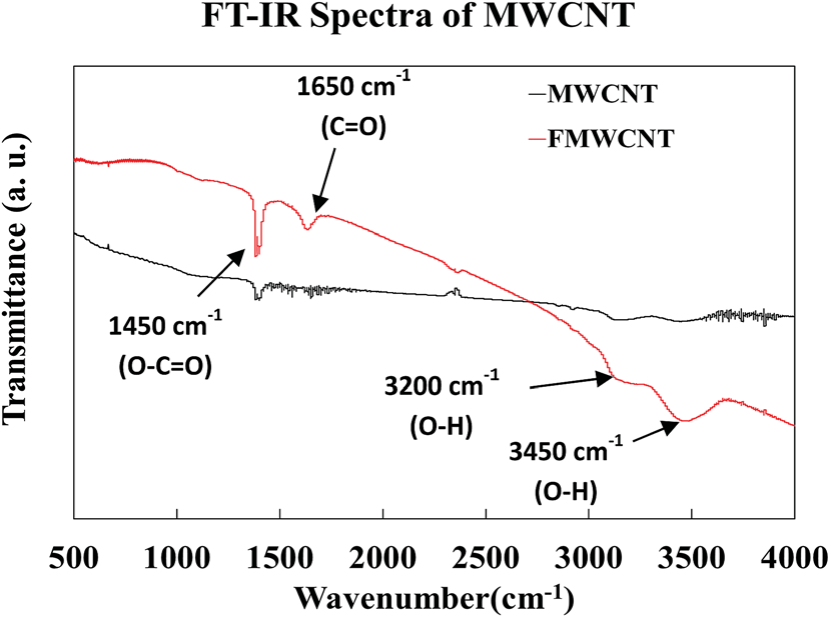
FT-IR spectra of the MWCNTs and FMWCNTs.

Fig. 11 shows the FT-IR spectra of both MWCNTs and FMWCNTs. In MWCNTs, the difference of charge states between carbon atoms is known to induce the formation of electric dipoles, when subjected to infrared radiation (IR).^54^ These dipoles can be detected as the IR spectra during the FT-IR analysis.^44,54^ In MWCNTs, the symmetrical structure results in a relatively silent spectrum as weak infrared signals are detected due to the weak difference of charge states between the symmetrical carbon atoms.^44^ However, in the FMWCNTs, multiple characteristic peaks were observed in the FT-IR spectra indicating the presence of functional groups that are introduced due to the acid treatment. Broad peaks between 3000 cm^−1^ and 3500 cm^−1^ were obtained indicating the characteristic stretching vibrations of hydroxyl (O–H) bonds.^44^ Further, the FT-IR spectrum of the FMWCNTs showed the peaks of carboxyl (C=O) and carbonyl (O–C=O) groups at ≈1650 cm^−1^ and ≈1450 cm^−1^, respectively.^44,55,56^ Thus, these peaks from the FT-IR indicated the presence of functional groups on the surface of FMWCNTs after covalent acid functionalization.

### 3.4 Dispersion analysis

The results of the dispersion analysis for the MWCNTs and FMWCNTs are shown in Fig. S4.† Vigorously stirred solutions of the MWCNTs and FMWCNTs (0.5 wt%) in DI water were held for up to 5 days to investigate their suspension stability. Sedimentation of the MWCNTs was observed, within 60 seconds, which demonstrated poor suspension stability. This sedimentation occurred due to the agglomeration of the MWCNTs, caused by van der Waal's forces of attraction.^44^ On the other hand, the FMWCNTs showed better suspension stability for up to 5 days, as no sedimentation was observed. This stability is due to the presence of the equally charged functional hydroxyl and carboxyl groups which enable the MWCNTs to repel each other and, thus, keep the solution dispersed.^44^

### 3.5 Humidity sensor characterization

**Fig. 12:**
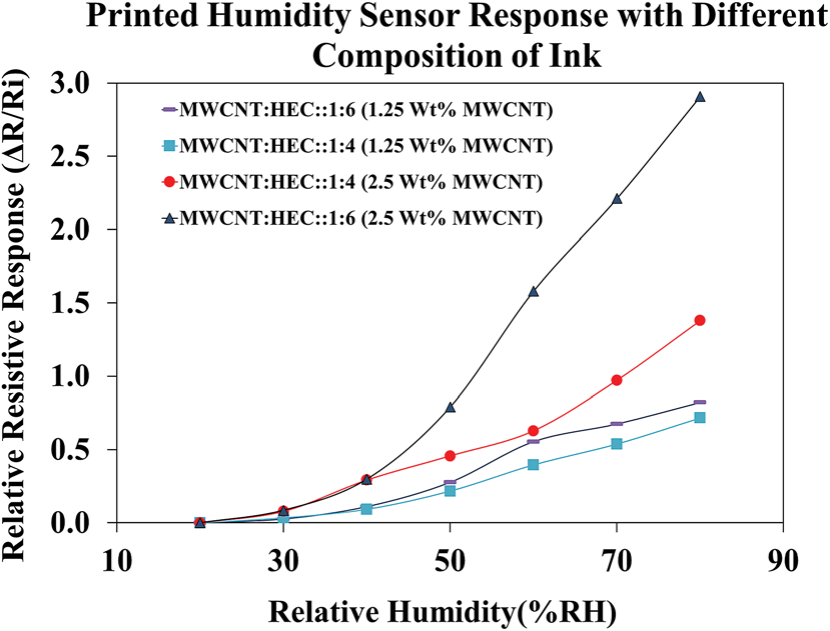
Response of the humidity sensor with different compositions of the FMWCNT/HEC ink.

Before subjecting the humidity sensors to varying humidity levels, the sensors were pre-heated on a VWR 810 ceramic hot plate at 90 °C for 1 hour to remove the water molecules trapped around the FMWCNTs and in between the electrodes. Fig. 12 shows the relative resistive response of printed humidity sensors fabricated using different ink compositions. The content of the FMWCNTs was varied from 1.25 wt% to 2.5 wt% in the ink, with respect to the solvent and the weight ratio between FMWCNTs and HEC was varied from 1 : 4 to 1 : 6. It was observed that the printed humidity sensor fabricated with 1.25 wt% FMWCNTs (FMWCNTs : HEC: 1 : 6) yielded a maximum relative resistive response of 0.82, at 80% RH. By maintaining the content of the FMWCNTs at 1.25 wt% and decreasing the ratio between the FMWCNTs and HEC to 1 : 4, the maximum relative resistive response of the sensor decreased to 0.71, at 80% RH. This decrease in maximum relative resistive response can be attributed to the lower HEC content. Further, with increasing the content of the FMWCNTs to 2.5 wt% and maintaining the ratio of FMWCNTs and HEC at 1 : 4, the maximum relative resistive response of the printed sensor increased to 1.37, at 80% RH. This is due to the availability of more FMWCNTs for the adsorption of water molecules on their surface. By elevating the ratio of the FMWCNTs and HEC to 1 : 6, the maximum relative resistive response of the printed sensor increased to a relatively higher value of 2.91, indicating that the relative resistive response of the printed humidity sensor can be tunable by varying the ink composition. The ink with 2.5 wt% FMWCNTs (FMWCNTs : HEC: 1 : 6), which had the highest maximum relative resistive response, was chosen for further evaluation in this study.

**Fig. 13:**
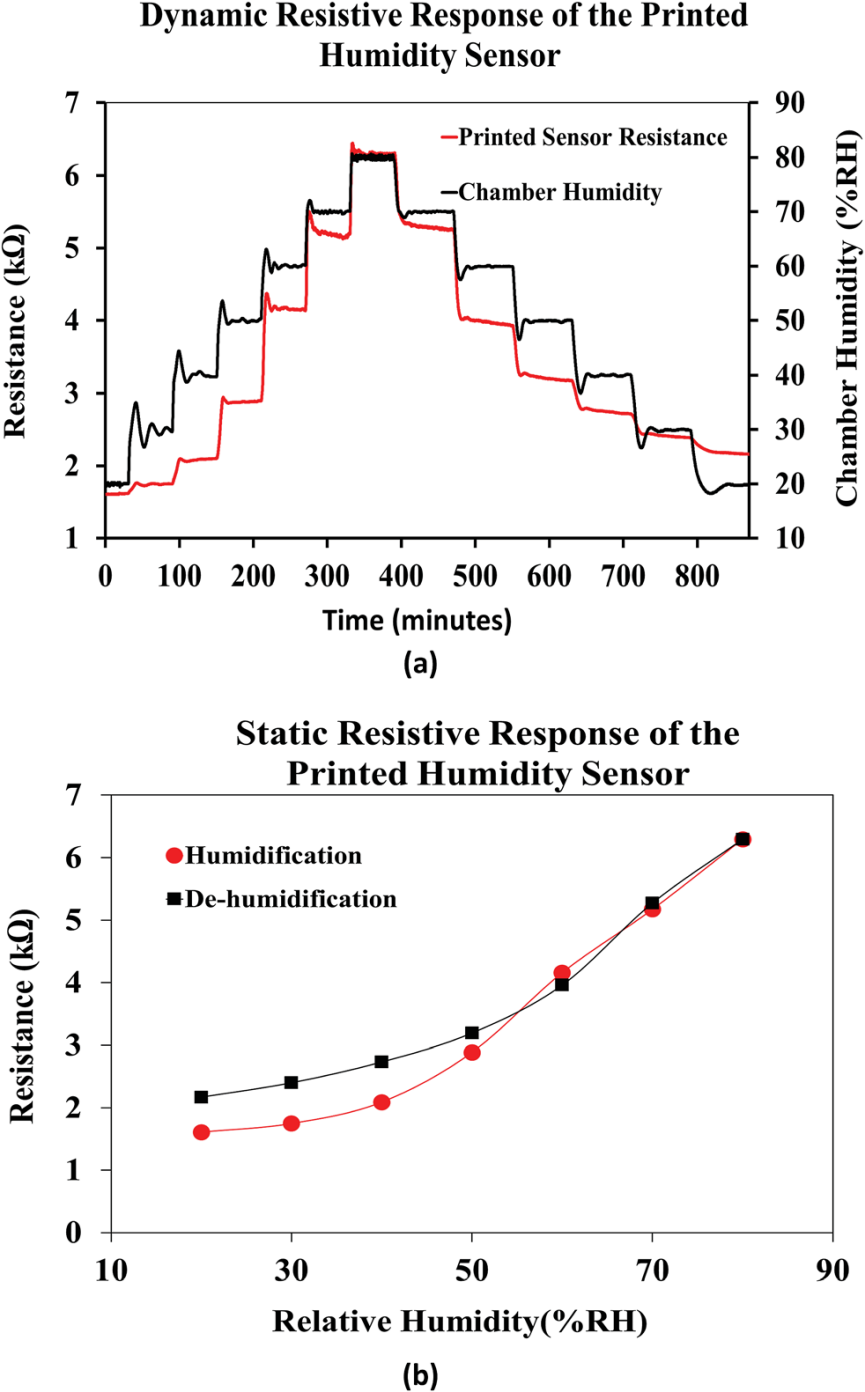
(a) Dynamic and (b) static response of the printed humidity sensor.

Fig. 13(a) shows the dynamic resistive response of the printed sensor towards varying relative humidity. The humidity of the chamber was varied form 20% RH to 80% RH during the humidification process and from 80% RH to 20% RH during the de-humidification process, in steps of 10% RH at a constant temperature of 25 °C. Accordingly, the resistance of the humidity sensor increased during the humidification process and recovered back during the de-humidification process, over time. The humidity sensing mechanism of the FMWCNT/HEC is based on the electron donation model of the MWCNTs and swelling behavior of the HEC. The MWCNT network exhibits p-type semiconducting characteristics, where the electrical conduction is dominated by holes.^57^ When exposed to humidity in the environment, the resistance changes in the MWCNTs depend on their interaction with water molecules. These water molecules are adsorbed onto the surface of the MWCNTs through physisorption, and tend to transfer electrons to MWCNTs due to the difference in electrical potential between them.^19,58^ This charge transfer mechanism decreases the number of holes and consequently increases the electrical resistance of MWCNTs. The RH level of the environment directly dictates the amount of water molecules to be adsorbed on the MWCNTs. As the RH level increases, more water molecules are adsorbed, and more electrons will be transferred, leading to a further increase in the resistance value.^11^ In addition, the hydroxyl and carboxyl groups present on the defective sites of the FMWCNTs show increased affinity towards water molecules (hydrophilicity). This improved hydrophilicity not only enhances the interaction between FMWCNTs and water molecules, but also aids in increasing the number of electrons transferred, contributing to a much higher resistance change.^11^ Further, the HEC consists of abundant hydroxyl groups and exhibits physical swelling upon exposure to humidity due to the adsorption of a large number of water molecules.^59^ This swelling property of HEC can effectively increase the contact gap of the FMWCNT intertube junction in the composite, thereby contributing to the increase in the resistance with humidification.^11^ However, during de-humidification the number of effective charge carriers (holes) in the FMWCNTs increases as the water molecules desorb from their surface.^64^ This increase in the number of effective charge carriers results in a decrease in the resistance values. In addition, HEC starts to physically shrink as the water molecules desorb from its surface.^65^ This phenomenon decreases the contact gap of the FMWCNT intertube junction in the composite, hence resulting in a decrease in the resistance during dehumidification.

Fig. 13(b) shows the average static resistive response of the printed humidity sensor. It was observed that during the humidification process, the resistance of the sensors increased from 1.6 kΩ to 6.3 kΩ, when the relative humidity in the chamber was varied from 20% RH to 80% RH. This resulted in an overall resistance change of ≈290% at 80% RH, when compared to the base resistance at 20% RH. A sensitivity (*S*) of 0.0485/%RH was mathematically calculated for the fabricated humidity sensor using eqn (1).1
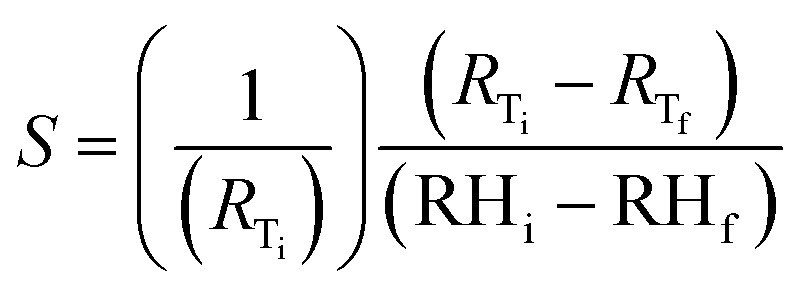
where *R*_T_i__ and *R*_T_f__ are the resistance values obtained at initial relative humidity RH_i_ and final temperature RH_f_. The results obtained for the printed humidity sensor demonstrated a sensitivity which is an order of magnitude greater than the sensitivity of a MWCNT network-based humidity sensor (0.005/%RH) reported by Liu *et al.*^19^ In addition, the sensitivity of the printed FMWCNT/HEC based humidity sensor was better than those of several other MWCNT and MWCNT/polymer-based humidity sensors reported^15,19,20,60–62^ and a comparison is shown in Table 1. It was observed that the printed humidity sensor exhibited a hysteresis effect at low moisture levels during humidification/de-humidification. This hysteresis can be attributed to the charge trapping by water molecules present around FMWCNTs, especially with their improved hydrophilicity.^66^ The trapped charge contributes to a comparatively higher resistance, during de-humidification when compared to the resistance, during humidification.

**Table 1:** Comparison of sensitivity between the printed FMWCNT/HEC humidity sensor and other reported printed sensors

Author	Materials	Sensitivity	Response time	Recovery time	Ref.
Liu *et al.* (2009)	MWCNT network	0.0056/% RH	3 s	25 s	19
Yu *et al.* (2006)	PEI/MWCNT film	0.0099/% RH	2 s	30 s	60
Cao *et al.* (2011)	Acid treated MWCNT film	0.0154/% RH	50 s	140 s	20
Yoo *et al.* (2010)	O_2_ plasma-treated PI/MWCNT film	0.0047/% RH	Not reported	Not reported	15
Tang *et al.* (2011)	PI/MWCNT film	0.0018/% RH	Less than 5 s	Not reported	61
Chu *et al.* (2013)	Carbon nanosheet based sensor	0.0268/% RH	30 s	90 s	62
Turkani *et al.* (2018)	Functionalized MWCNT/HEC	0.0485/% RH	≈20 s	≈35 s	Present work

**Fig. 14:**
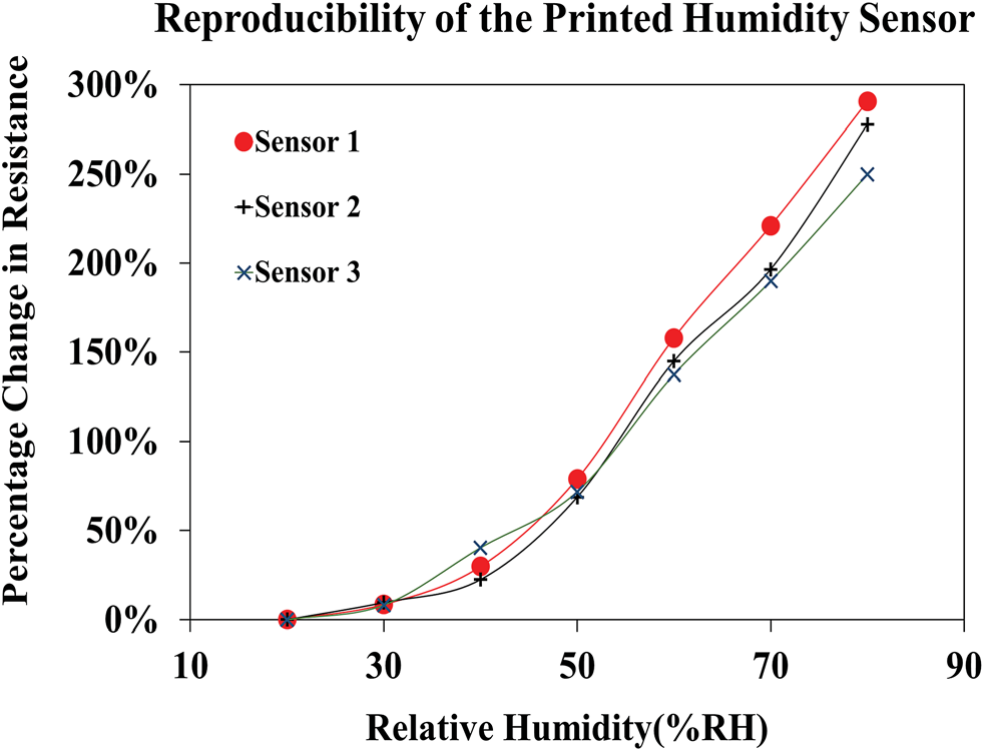
Reproducibility of the humidity sensor.

The reproducibility of the sensor performance was investigated by analyzing the resistive response of three different sensors printed using the FMWCNT/HEC ink, formulated separately for each sensor (Fig. 14). It was observed that all three fabricated sensors demonstrated a similar response towards relative humidity varying from 20% RH to 80% RH, yielding an average maximum resistance change of 273% ± 21% at 80% RH, when compared to base resistance at 20% RH. The obtained results prove the reproducibility of both the FMWCNT/HEC ink and the printed humidity sensor.

**Fig. 15:**
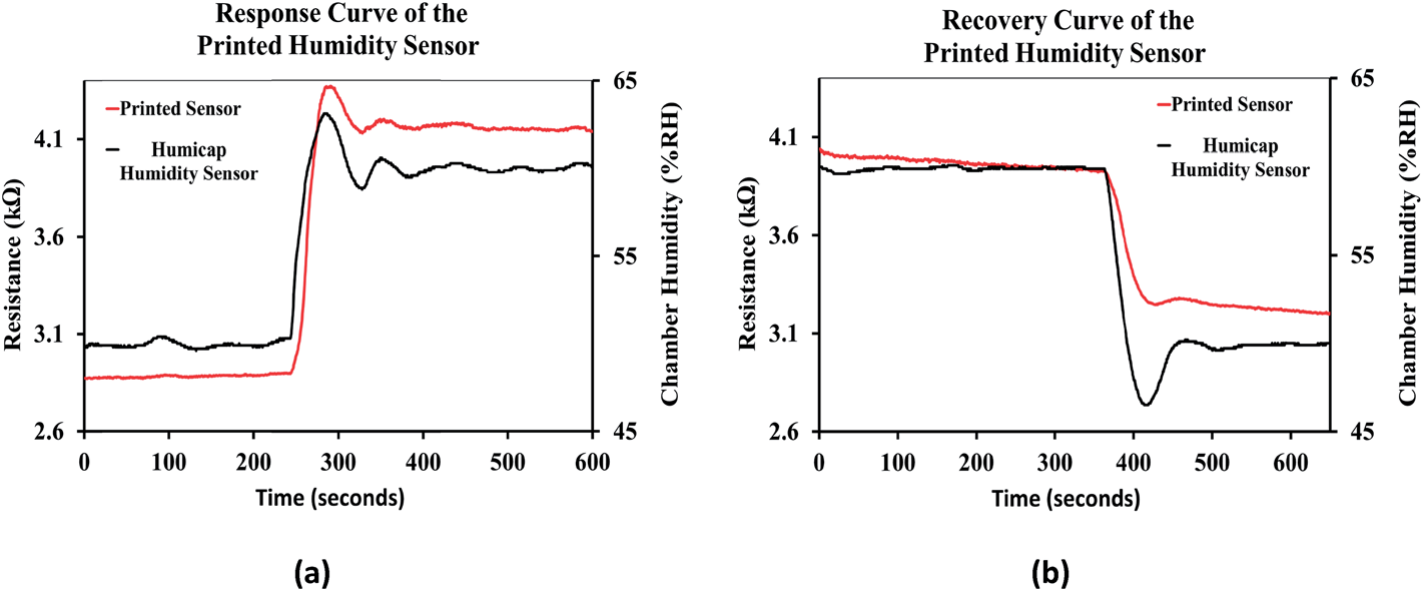
(a) Response curve and (b) recovery curve of the printed humidity sensor.

The response curve of the printed humidity sensor is shown in Fig. 15(a). It was observed that the resistive response of the printed sensor followed the response of the integrated humidity sensor (HUMICAP 180, Vaisala) in the environmental chamber, when the relative humidity of the chamber was varied from 50% RH to 60% RH. Since the response time of the integrated humidity sensor is less than 20 seconds, the response time of the printed humidity sensor was estimated to be ≈20 seconds and is comparable with the performance of conventional impedance type humidity sensors, which typically ranges from 10 seconds to 30 seconds.^6,63^ The recovery curve of the printed humidity sensor when the relative humidity of the chamber was varied from 60 %RH to 50 %RH is shown in Fig. 15(b). The recovery time of the printed humidity sensor was estimated to be ≈35 seconds and is also comparable with those of conventional impedance type humidity sensors, which typically range from 15 seconds to 35 seconds.^6^

**Fig. 16:**
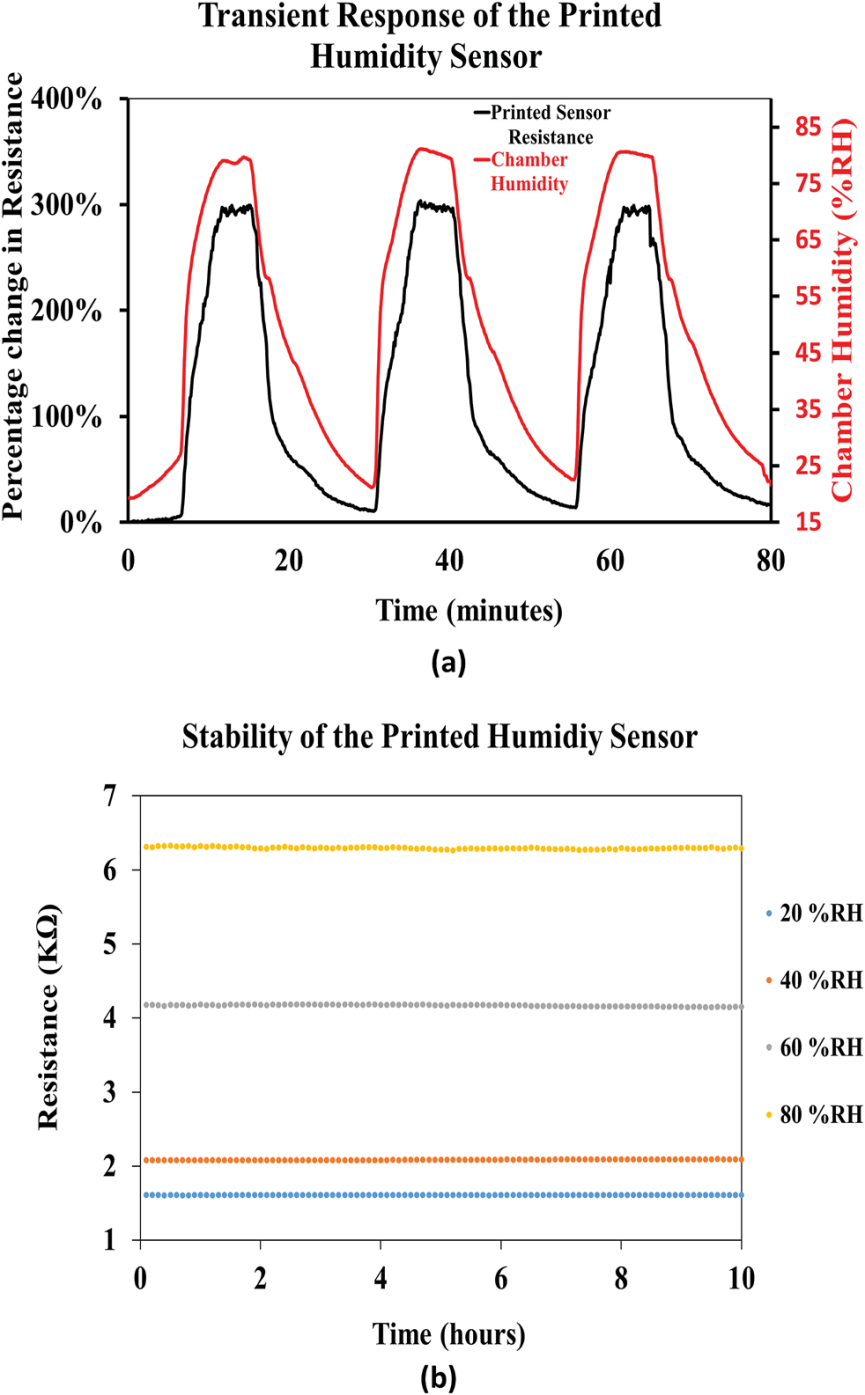
(a) Transient response of the printed humidity sensor and (b) stability of the printed humidity sensor.

Fig. 16(a) shows the transient response of the printed humidity sensor demonstrating dynamic humidification and de-humidification cycles. The relative humidity in the chamber was interchanged between 20% RH and 80% RH, at a constant relative temperature of 25 °C, and the response of the printed sensor followed the interchangeability with increase and decrease in the resistance values. Throughout this experiment, the printed sensor yielded a consistent maximum resistive change of ≈290%, thus demonstrating the repeatability of the printed sensor. In addition, the stability of the printed humidity sensor, over time, was investigated by recording its resistive response at different relative humidities (20% RH, 40% RH, 60% RH, and 80% RH), for a period of 10 hours (Fig. 16(b)), at a constant chamber temperature of 25 °C. A drift of 0.10%, 0.16%, 25%, 0.28% and 0.21% in the resistance was observed for a constant relative humidity of 20% RH, 40% RH, 60% RH, and 80% RH, respectively. It was observed that the printed humidity sensor was capable of sensing a broad range of relative humidities (20% RH to 80% RH) while demonstrating good stability over time. From the results obtained, it is evident that the printed humidity sensor has the capability to be employed for humidity sensing applications in the automobile, laboratory and food industries.

## 4. Conclusion

In this work, a fully printed, highly sensitive, humidity sensor based on a FMWCNT and HEC composite was successfully developed for humidity monitoring applications. To enhance the hydrophilicity, MWCNTs were treated in a mixture of sulfuric and nitric acid. Transmission electron microscopy, Raman spectroscopy, Fourier transform infrared spectroscopy and dispersion analysis were performed to confirm the functionalization and the presence of functional groups on the surface of the MWCNTs. A FMWCNT/HEC composite ink was formulated with 2.5 wt% MWCNTs and the ratio between FMWCNTs and HEC was maintained at 1 : 6. A pair of electrodes with 24 fingers in an interdigitated geometry were deposited using the screen printing process. The formulated FMWCNT/HEC ink was deposited on the electrodes as the sensing layer using the gravure printing process. The performance of the printed humidity sensor was investigated by measuring its resistive response towards relative humidity varying from 20% RH to 80% RH, in steps of 10% RH, at a constant temperature of 25 °C. The printed sensor exhibited resistive changes as high as 290% at 80% RH, when compared to its base resistance at 20% RH with a sensitivity of 0.048/%RH. The obtained sensitivity of the sensor was greater than those of several other MWCNT and MWCNT/polymer based humidity sensors reported. The reproducibility in terms of ink formulation and fabrication of sensors was also investigated. In addition, the printed humidity sensor demonstrated a good response time of ≈20 s when measured against a commercial humidity sensor equipped in the environmental chamber.

These results demonstrate that humidity sensors fabricated using additive print manufacturing processes on flexible substrates, with the FMWCNT/HEC composite as the sensing material, have a significant potential in humidity sensing applications. Moreover, the printed humidity sensor can potentially be implemented in applications where its lightweight and conformal features along with large scale manufacturing capabilities are paramount for improved user–device interactions. Further research is underway to investigate the performance of the printed humidity sensor for varying temperatures along with the compensation strategies. Research is also being focused on the monolithic integration of a printed micro-heater with the humidity sensor and, thereby, the improvement of the reliability of the sensor by reducing the hysteresis. Finally, the effect of mechanical stresses such as bending, twisting, and stretching on the performance of the sensor is also under investigation to determine appropriate output compensation required, before implementing the printed humidity sensor into a field deployable sensing system.

## Conflicts of interest

There are no conflicts to declare.

## Supplementary Material

NA-001-C9NA00179D-s001
