# Supplementary material for: A highly sensitive printed humidity sensor based on a functionalized MWCNT/HEC composite for flexible electronics application
Source: Nanoscale Adv. 2019 Apr 15;1(6):2311–22. doi: 10.1039/c9na00179d (PMC9419184; doi:10.1039/c9na00179d)
Supplement: NA-001-C9NA00179D-s001 [file NA-001-C9NA00179D-s001.pdf]

Electronic Supplementary Material (ESI) for Nanoscale Advances.
This journal is © The Royal Society of Chemistry 2019

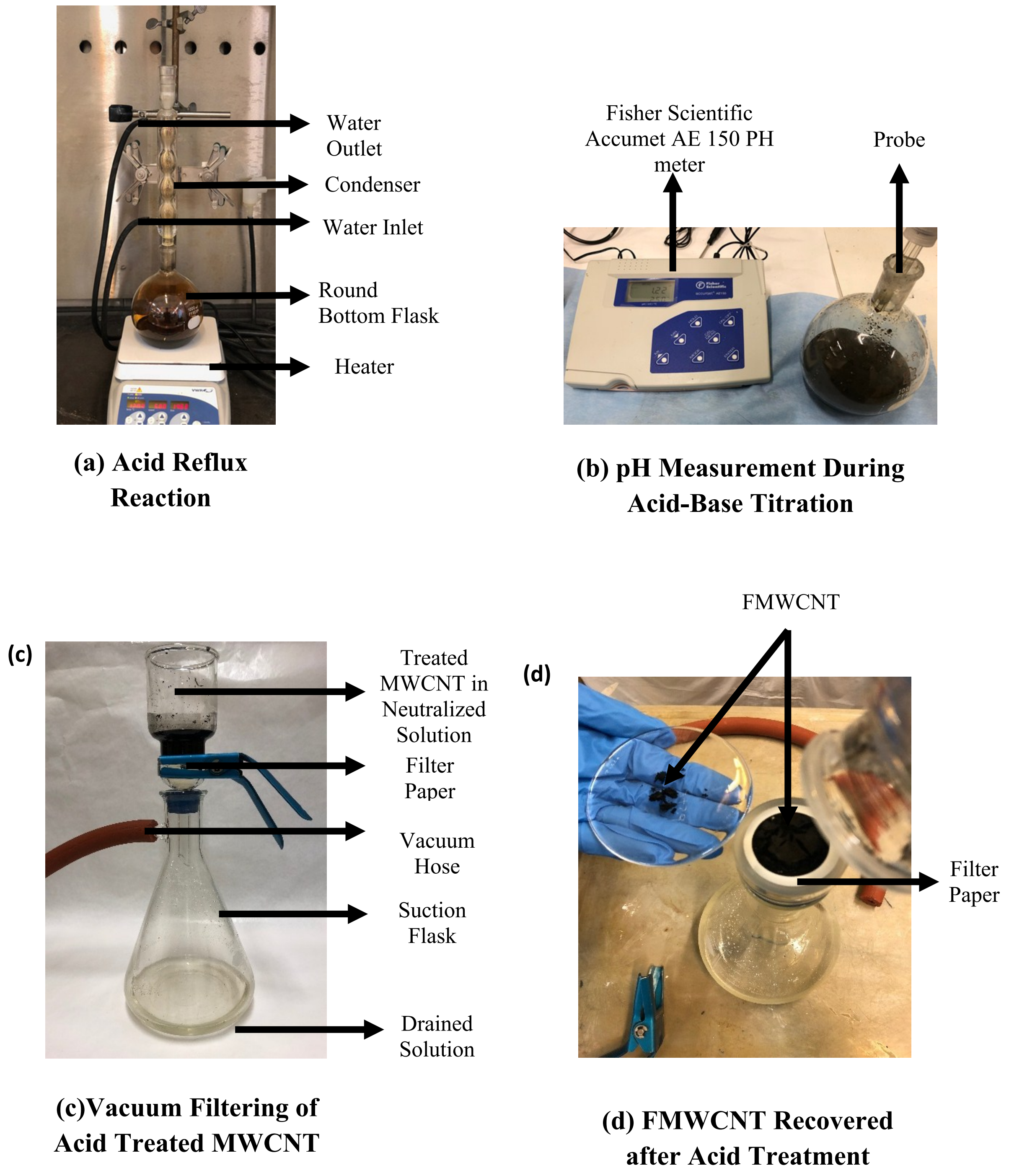


**Figure S1.** Functionalization of MWCNT (a) acid reflux reaction, (b) pH measurement during acid-base titration, (c) vacuum filtering of acid treated MWCNT and (d) FMWCNT recovered after acid treatment.

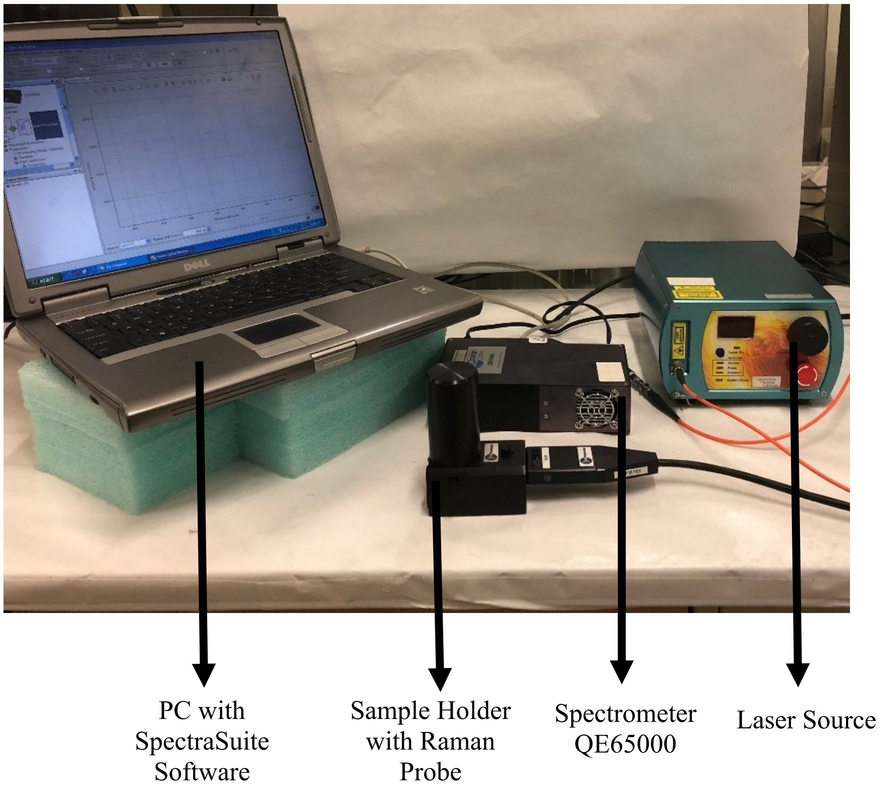


**(a)**

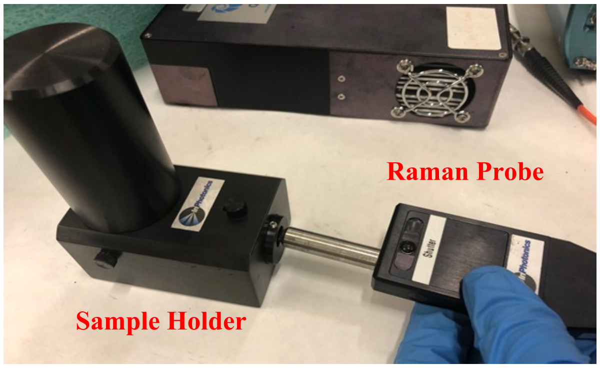


**(c)**

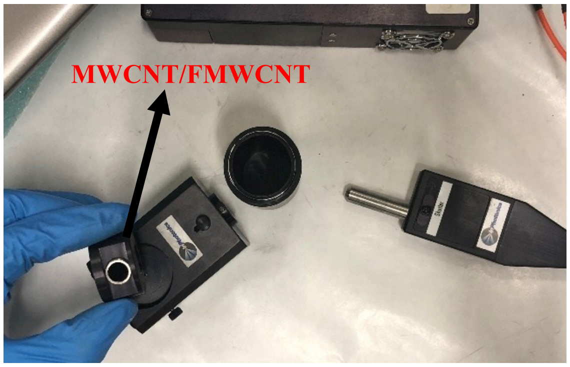


**(b)**

**Figure S2.** Raman spectroscopy of MWCNT and FMWCNT: (a) experiment setup, (b) Raman probe and the sample holder, and (c) MWCNT/FMWCNT in sample holder.

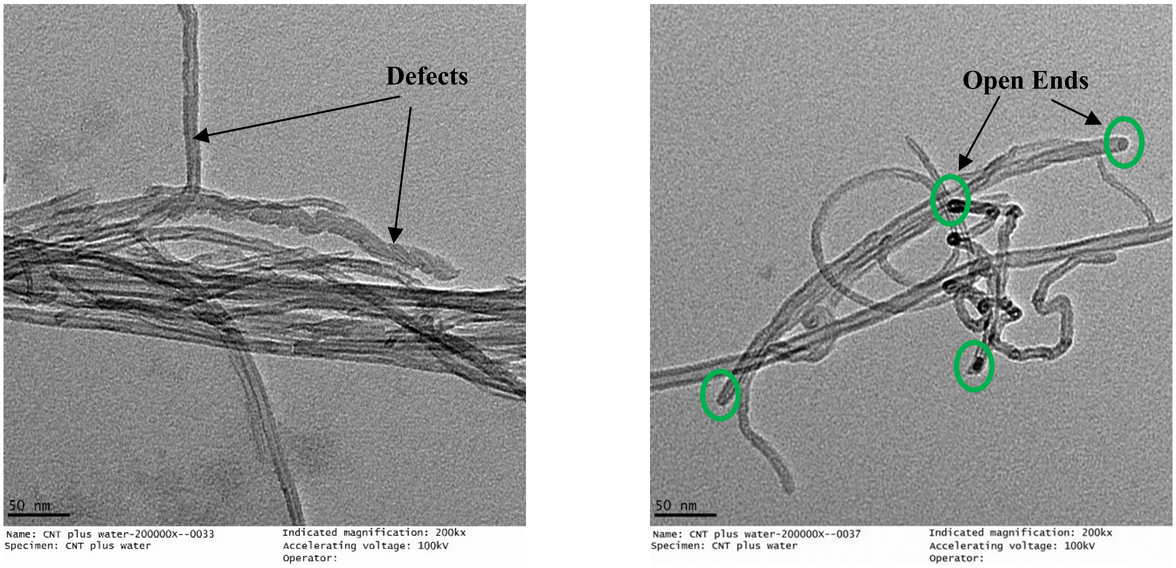


(a) (b)

**Figure S3.** TEM micrographs of FMWCNT showing (a) defects on the sidewalls and (b) open ends.

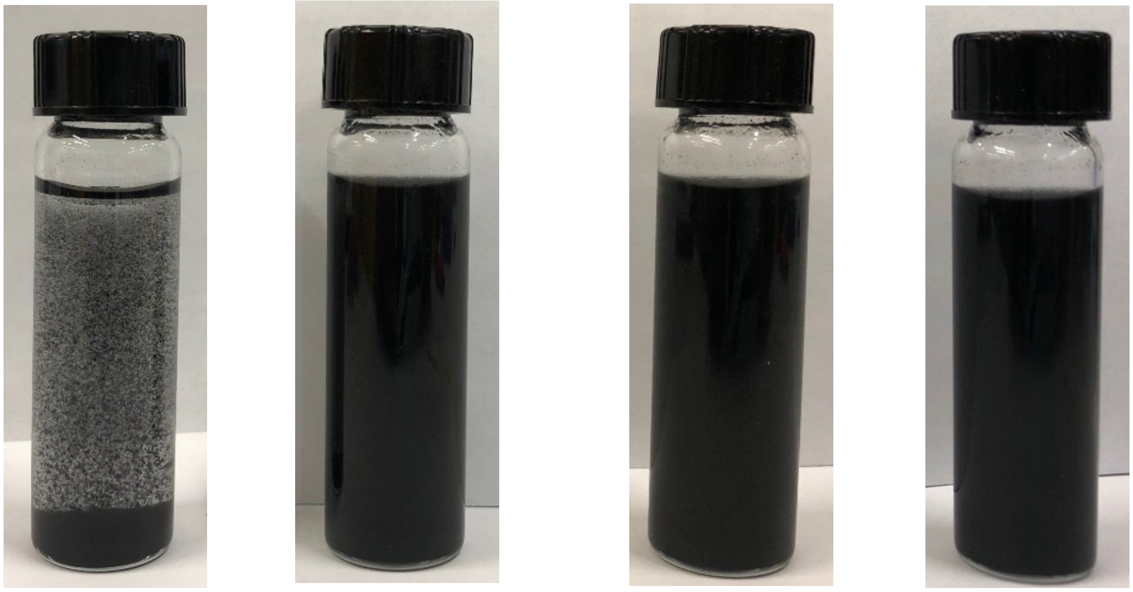

**MWCNT (60 seconds)** | **FMWCNT (60 seconds)** | **FMWCNT (After 72 hours)** | **FMWCNT (After 120 hours)**

**Figure S4.** Dispersion analysis of MWCNT and FMWCNTs for upto 5 days.
